# Long lifespan and substantial biomass production support stable high biomass of *Ascophyllum nodosum* under interannual climate fluctuations in Greenland

**DOI:** 10.1111/jpy.70071

**Published:** 2025-08-25

**Authors:** Birgit Olesen, Núria Marbà, Carlos M. Duarte, Dorte Krause‐Jensen

**Affiliations:** ^1^ Department of Biology Aarhus University Aarhus Denmark; ^2^ Arctic Research Centre, Aarhus University Aarhus Denmark; ^3^ Global Change Research Group, Institut Mediterrani d'Estudis Avançats (CSIC‐UIB) Esporles Spain; ^4^ Red Sea Research Center, King Abdullah University of Science and Technology (KAUST) Thuwal Saudi Arabia; ^5^ Department of Ecoscience Aarhus University Aarhus Denmark

**Keywords:** Arctic, foundations species, macroalgae, nutrients, populations dynamics, recruits, warming

## Abstract

The brown macroalga *Ascophyllum nodosum* is a foundation species on intertidal rocky shores, where its perennial canopy and high productivity support key ecological functions. However, its population dynamics near the northern edge, where low temperatures and sea ice may challenge stability, are largely unknown. We followed the population structure, dynamics, and nutrient status of *A. nodosum* in the sheltered, subarctic Kobbefjord, Greenland from 2012 to 2019. Despite the northern location, population biomass (16–27 kg FW · m^−2^) was within the upper known range and was dominated by few large (max length: 109 cm), old individuals (observed age: up to 19 years; estimated mean lifespan: 37.5 years based on intrinsic mortality rate). Population density remained stable because of low mortality (0.019 · year^−1^) and recruitment rates (0.010 · year^−1^), sustained by an understory of small juveniles. Biomass increased 1.5‐fold over the 8‐year study, supported by high biomass productivity (3.3–8.1 kg FW · m^−2^ · year^−1^) that balanced branch loss and reflected a moderate biomass turnover time (2.6–6.3 years) of organic matter, underlying the apparent stability. Such overall population stability reflects a “biomass storer” strategy typical of environments with low disturbance and nutrient levels. The stability is remarkable given seasonal ice cover (2–7.5 months per year), large variation in average daily temperature (−3.9 to 15.4°C), and low nutrient supply. While ice breakup poses a risk of shoot abrasion, the ice cover likely provides protection against ice scouring. Enhanced growth during warmer summers and earlier ice break‐up suggests faster turnover rates in the future to the extent nutrient availability can support it.

**Abbreviations**
ANOVA: analysis of variance
*C*_base_
: circumference above holdfast
*C*_max_
: maximum circumference of individual
HSD: honestly significant difference
*L*
: maximum length of individual
M: mortality rate
*N*
: initial number of individuals
*N*_B_
: new individuals
*N*_surv_
: surviving individuals
*r*
: net population growth rate
*R*
: recruitment rate
*t*_D_
: population doubling or halving time

## INTRODUCTION

Macroalgal habitats fringe intertidal and shallow subtidal coastlines around the globe, in which they are key primary producers and canopy formers, supporting carbon and nutrient fluxes, biodiversity, and coastal fisheries (Christie et al., 2009; Duarte et al., 2022; Pessarrodona et al., 2022; Teagle et al., 2017). These ecosystem functions are closely linked with the structure and dynamics of the habitats.

Foundation species among macroalgae are often canopy‐forming brown macroalgal genera, such as *Ascophyllum* and *Fucus* in the tidal zone and kelps such as *Laminaria, Saccharina*, and *Alaria* in the subtidal (Filbee‐Dexter et al., 2019; Schmidt et al., 2011). Many of those species have broad geographical distribution ranges extending into the Arctic region, despite having temperate origins (Müller et al., 2009; Wulff et al., 2009). Although several of the species can develop high biomass even in the Arctic (Filbee‐Dexter et al., 2022; Ørberg et al., 2018; Thyrring et al., 2021), their growth may be constrained by low temperatures, ice cover, and the associated light reduction and physical scouring, limiting both intertidal macroalgal species (Marbà et al., 2017) and subtidal kelps (Krause‐Jensen et al., 2012) relative to further south. The same is true for the only seagrass extending to the Arctic region, *Zostera marina* (Olesen et al., 2015). However, there is very limited information on the population dynamics of macroalgal foundation species in Polar Regions. This is a critical knowledge gap because biomass and population turnover affect key ecosystem functions, and the extreme climatic conditions of the polar region might compromise habitat stability and, thereby, functionality.

In Greenland, the rocky intertidal communities are often dominated by the canopy‐forming macroalgae *Ascophyllum nodosum* (knotted wrack), especially in sheltered settings, and *Fucus* spp. (Høgslund et al., 2014; Ørberg et al., 2018; Sejr et al., 2021; Thyrring et al., 2021). *Ascophyllum nodosum*, like other fucoid canopy formers, creates key habitats that support complex food webs and biodiversity, while modifying environmental conditions (Christie et al., 2009; Coleman & Wernberg, 2017; Josselyn & Mathieson, 1978; Pereira et al., 2020). Its long‐lived, dense canopy protects intertidal fauna from extreme temperatures, such as those in the sub‐Arctic, and facilitates colonization (Ørberg et al., 2018). Additionally, *A. nodosum* contributes to carbon and nitrogen storage in its biomass, a fraction of which may potentially be exported to marine carbon sinks beyond its habitat (Ager et al., 2023; Schmidt et al., 2011). Economically, *A. nodosum* is important along rocky shores of the North Atlantic, harvested for use in agriculture and animal feed (Pereira et al., 2020).

*Ascophyllum nodosum* is particularly well suited for studying macroalgal population structure and dynamics in the Arctic, where fieldwork time is often limited. This is because *Ascophyllum* is a long‐lived modular macroalga that adds a new bladder every year through apical growth, resulting in distinct segments of increasing age from the tip to the base of its long, unbroken shoots, thereby allowing reconstruction of age structure and annual growth increments of individuals from their morphology. When combined with individual tagging, this makes it possible to study population dynamics through annual plot revisits. Although the growth rates of *A. nodosum* individuals in Greenland have increased with warming (Marbà et al., 2017), the dynamics at the population level in terms of recruitment, mortality, and net population change are unknown. Filling this knowledge gap will increase the understanding of drivers of losses and gains and provide a better basis for predicting and supporting the resilience of these communities.

Genetic surveys have suggested that *Ascophyllum nodosum* survived the last glacial maximum on both sides of the Atlantic, documenting a large capacity to persist (Olsen et al., 2010). *Ascophyllum* individuals can also grow old, with maximum lifespans estimated at 50–60 years (based on extrapolation of data on cohort survival), and genets may be older because the basal plate can keep forming new shoots of the same individual to replace old ones (Åberg, 1992a). Population dynamics of *A. nodosum* have been explored at the equatorial edge of the geographical distribution range (Portugal, Araújo et al., 2014; Viana et al., 2014) as well as centrally in the distribution range, for example, in Sweden (Åberg, 1992a, 1992b), the Irish Sea (Svensson et al., 2009), France (Araújo et al., 2014), and Maine, United States (Dudgeon & Petraitis, 2005), but not under sub‐Arctic or Arctic settings. The general pattern has been relatively stable populations of high biomass dominated by large, old individuals with low mortality. In line with this, populations in Brittany, France, have been characterized as “old growth forests” with little recruitment, making them susceptible to disturbances such as harvesting and ice scour (Olsen et al., 2010, p. 853). The extent to which similarly structured populations occur in the northern part of the distribution range is unknown. As sub‐Arctic and Arctic *A. nodosum* populations can develop high biomass (Ørberg et al., 2018; Sejr et al., 2021; Thyrring et al., 2021) despite relatively low growth rates (Marbà et al., 2017), they are likely to form even older populations than those further south, which might also be susceptible to impacts such as those generated by the extreme climatic conditions.

This study examined the population dynamics of *Ascophyllum nodosum* in sub‐Arctic conditions. The study site, located at 64° N in the Nuup Kangerlua fjord system on Greenland's west coast, lies five latitude degrees south of the species' polar distribution limit in Greenland (69° N; Pedersen, 2022) but is still exposed to seasonal ice cover, potential ice scouring, short winter days, and large seasonal temperature fluctuations. The study was based on 8 years of annual monitoring of *A. nodosum* in the inner, sheltered part of Kobbefjord under the Greenland Ecosystem Monitoring program, supplemented by additional data from other *Ascophyllum* populations in the vast Nuup Kangerlua fjord system. Data were related to sea ice cover, temperature, and tissue nutrient content. Because of Ascophyllum's boreal origin and temperature optimum of 15–20°C (Fortes & Lüning, 1980; Wilson et al., 2015), which is considerably above sub‐Arctic averages, we hypothesized that northern *A. nodosum* populations exhibit slower rates of annual biomass production and shoot recruitment than those further south, potentially increasing their sensitivity to environmental disturbances.

## MATERIALS AND METHODS

### Study site

**FIGURE 1:**
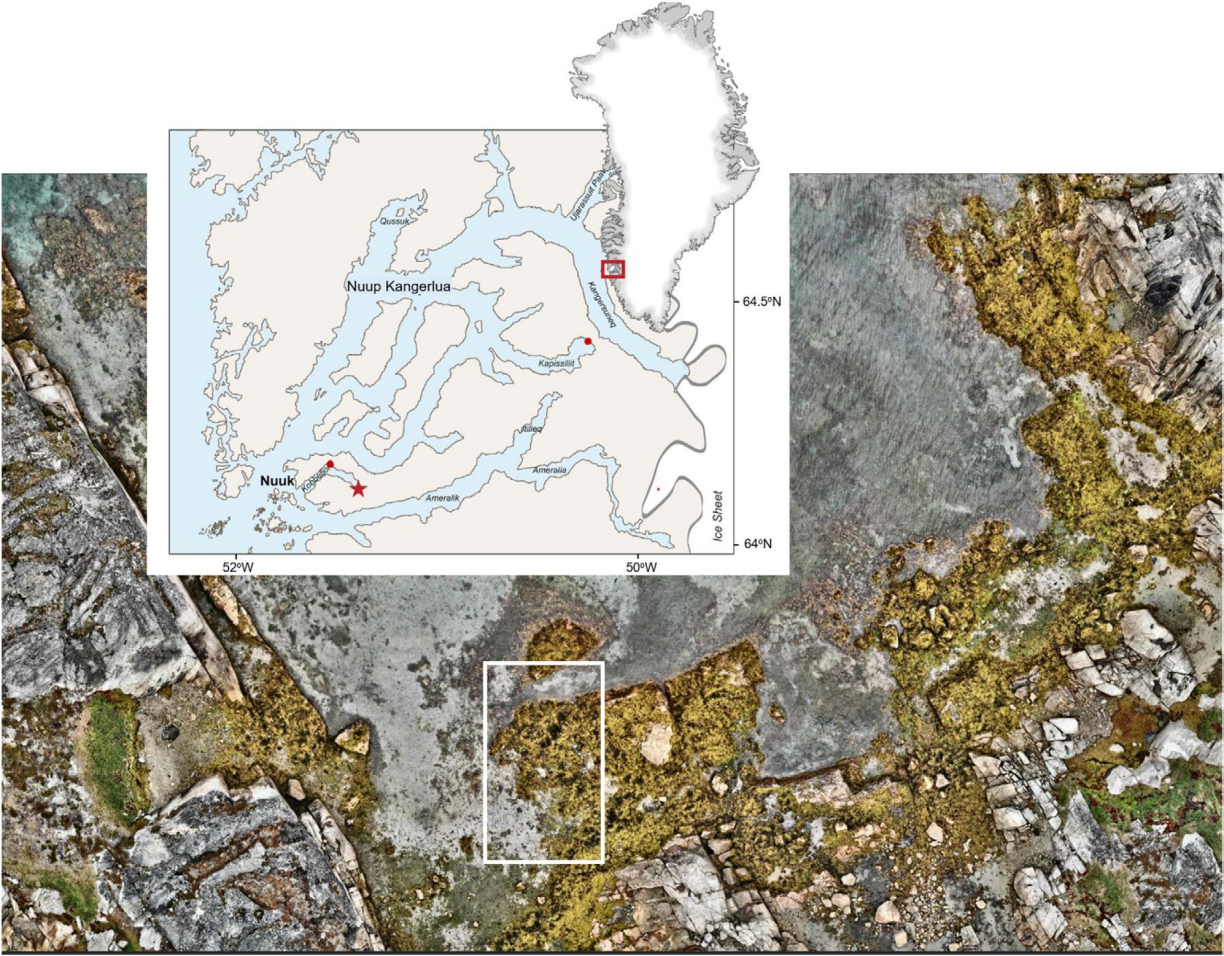
Map of the Nuup Kangerlua fjord system, SW Greenland, and low‐tide drone image of the study site (red star, white rectangle) located in the inner part of Kobbefjord. Supplementary biomass sampling sites in inner‐ and central Kobbefjord and Kapisillit are marked by red dots.

The study took place in the Nuup Kangerlua fjord system in southwest Greenland, where the rocky intertidal of many inner fjord branches is dominated by the canopy‐forming fucoid, *Ascophyllum nodosum* (Figure 1). The study site was in the inner sheltered part of Kobbefjord (64^o^08′ N, 51^o^23′ W). Three supplementary *A. nodosum* sites were also included, representing other sheltered locations within the system: one in inner Kobbefjord (150–200 m from the main study site), one in central Kobbefjord (64°10′ N, 51°29′ W), and one in Kapisillit (64°28′ N, 50°13′ W; Figure 1). The main study site targeted *A. nodosum* population dynamics whereas the supplementary sites expanded the dataset on *A. nodosum* density, biomass, and morphology and were used to establish morphology‐biomass relationships (see details below).

Kobbefjord is 17 km long, 0.8–2 km wide, and has a maximum depth of 150 m. It is characterized by large variations in key physical parameters. The tidal range is 1–5 m (Richter et al., 2011). Sea surface temperatures range from −1 to 9°C (Versteegh et al., 2012), and air temperatures range from −25 to 20°C (measured at Nuuk, Blicher et al., 2013). Freshwater run‐off from a river in the inner Kobbefjord reduces the salinity of the study site during part of the year, and sea ice usually covers the inner fjord from December to May (Mikkelsen et al., 2008).

### Physical variables

Ambient temperature within the *Ascophyllum nodosum* canopy at the main study site was continuously recorded every 15 min from 2009 to 2019 with a Hobo Pendant logger. Daily average temperatures were calculated, and annual median values were used to represent the central tendency, as the median is less influenced by short‐term extremes and, therefore, better reflects typical conditions experienced by *A. nodosum* than the annual average temperature. To compare extreme temperature events between years, annual 1st and 99th percentiles were calculated.

The annual duration of sea ice cover at the study site was determined using photographs of the inner Kobbefjord taken daily at noon by a camera mounted at 500 m height on the mountain south of the inner fjord, facing north over the fjord. The camera was operated by the Greenland Ecosystem Monitoring program (https://doi.org/10.17897/4F6C‐XJ50; Figure S1). The first and last day of ice cover were identified for each year between 2012 and 2019.

Ice scouring was assessed using 10 “ice sensors” (screws) inserted in the rock at the lower right corner of each of 10 macroalgal plots (see below), and their divergence from the vertical axis after 1 year indicated whether there was ice scouring or not between visits. Bent screws were replaced at each visit. The scouring scale ranged from 0 to a maximum of 1, representing the case when all 10 screws were bent.

### *Ascophyllum* population structure and dynamics

#### Field set‐up

Population structure and dynamics of *Ascophyllum nodosum* were quantified at the main study site in the mid‐intertidal zone. Ten permanent plots (0.25 × 0.25 m) were randomly placed within the dense belt of mid‐intertidal *A. nodosum* vegetation (Figure 1). Each plot was marked by a stainless “eye”‐marker in the upper left corner and an “ice sensor screw” in the lower right corner. The distance between neighboring plots was 2–4 m.

The plots were established August 22, 2012, and revisited annually in late August or early September until 2019 (sampling dates varied from August 16 to September 9 between years). At the initiation of the survey, all individuals longer than 10 cm within the plots were tagged with numbered cable ties, affixed to their bases. Individuals were only included if most of their biomass was inside the plot. An individual represented one to several shoots, arising from a common basal holdfast, and all measurements were done at the level of the individual. All tagged individuals were defined as adults. The remaining individuals, all smaller than 10 cm, were considered a pool of juveniles and were not tagged because they were too small to retain the standard tags. Additionally, the limited time available for the annual survey at this remote location prevented the use of more sophisticated marking techniques.

#### Density, size, mortality, recruitment, and net population growth

Annual monitoring was nondestructive. We counted and measured the dimensions (length and circumference, see subsequent sections) of the marked individuals (>10 cm) and counted the untagged juveniles in two size classes: 2–10 cm and <2 cm. Juveniles were divided into the two size classes because 2 cm was the maximum length of recruits settled in bare plots after 1 year (Ørberg et al., 2018). The repeated counts of juveniles were used to estimate net change over time.

During each monitoring event, the number of surviving adult individuals and the appearance of new individuals longer than 10 cm (i.e., juveniles that had grown in length since the last census) were counted. Newly identified individuals exceeding 10 cm were tagged and numbered. This enabled the estimation of intrinsic rates of annual adult mortality (*M*), recruitment (*R*) of juveniles >10 cm, and the net change in the number of adult individuals (*r*). Due to limited demographic data, specifically the lack of stage‐specific fecundity and information on the fate of newly established recruits, it was not possible to construct life tables or develop population growth models. Therefore, overall population growth (or decline) was calculated directly from the annual counts of adult individuals (Silwertown & Charlesworth, 2001).

The annual mortality rate (*M*, year^−1^) was calculated as the exponential rate of loss of individuals between consecutive censuses:

(1) $$M=\frac{\ln\left(\frac{N_{0}}{N_{s\mathrm{urv}}}\right)}{t}\times 365$$

where *N*_0_ is the number of marked individuals in the plots at time *t*_0_, and *N*_surv_ is the number of surviving individuals at the next visit after *t* days. Assuming a constant mortality rate over time, the individual half‐life (*t*_½_, in years) was estimated as:

(2) $$t_{½}=\frac{\ln\left(2\right)}{M}$$

The annual recruitment rate (*R*, year^−1^) was estimated based on the number of marked individuals at *t*_0_ (*N*_0_) and the number of new individuals larger than 10 cm (*N*_B_) appearing between consecutive censuses:

(3) $$R=\frac{\ln\left(N_{0}+N_{B}\right)-\ln\left(N_{0}\right)}{t}\times 365$$

The annual net population growth rate (*r*, year^−1^) during the period (*t* days) between two annual censuses was calculated as:

(4) $$\;r=\frac{\ln\left(N_{t}\right)-\ln\left(N_{0}\right)}{t}\times 365$$

Finally, the population doubling time (*t*_D_, year^−1^; if *r* > 0) or halving time (if *r* < 0) was estimated as:

(5) $$t_{D}=\frac{\ln\left(2\right)}{r}$$

#### Age determination

At each annual visit, a minimum age of each marked individual was assessed by counting the number of consecutive bladders from the tip to the base of the longest shoot and adding 1 year, since one level of bladders is formed annually beginning in year 2 (Baardseth, 1970). Annual bladder formation has also been documented for Greenland populations (Marbà et al., 2017), but it may take more than 1 year for recruits to form their first bladder at the study site due to slow growth (2 cm · year^−1^; Ørberg et al., 2018). As most primary shoots break at some point and regrow from older parts, bladder counts provide only a minimum age estimate.

#### Biomass and minimum estimate of annual biomass production

For nondestructive estimation of *Ascophyllum nodosum* biomass in the permanent plots, we used size‐biomass relationships developed from individuals harvested in the Nuup Kangelua fjord system (see below). Accurate biomass predictions for *A. nodosum* have previously been achieved using relationships between the logarithm of weight and a proxy for volume, calculated as $Lc_{\max}^{2}$, where *L* is maximum length and *c*_max_ is the maximum circumference of the thallus (Åberg, 1990). A similar approach was applied by Gendron et al. (2018) for individuals trimmed to fixed lengths (15 and 30 cm) simulating harvest, with circumference measured 3–5 cm above the holdfast (*c*_base_). In this study, four size‐biomass relationships: $Lc_{\max}^{2}$, $Lc_{\text{base}}^{2}$, *Lc*_base_ and *L* alone, were developed using data from 217 *A. nodosum* individuals harvested in August 2011 and August 2012. Samples were collected from 23 quadrats measuring 0.15 × 0.15 cm and 10 quadrats measuring 0.25 × 0.25 cm, located at two sites in inner Kobbefjord (the study site and another site 150 m away) as well as one site in central Kobbefjord and one in Kapisillit (Figure 1).

For each individual, we recorded fresh weight biomass (*B*), length (*L*) from the holdfast to the tip of the longest shoot, base circumference (*c*_base_) 2–3 cm above the holdfast (capturing all basal shoots), and maximum circumference (*c*_max_). The coefficient of variation (*R* ^2^) was used to evaluate which model had the best predictive power (Appendix S1: Table S1). Although the model using $Lc_{\max}^{2}$ had the highest predictive power (*R* ^2^ = 0.981), we selected the simpler model based on *Lc*_base_ (*R* ^2^ = 0.901) to estimate annual biomass in the permanent plots (Appendix S1: Figure S2). This choice was made because *c*_base_ could be measured with minimal disturbance to the vegetation, which was often entangled in byssus threads. In years with heavy mussel settlements, it was difficult to separate the shoots without causing breakage, which could lead to loss of biomass. Moreover, attempts to measure the widest circumference (*c*_max_) in the presence of mussels led to overestimation. To reduce this bias, we measured circumference at a fixed height above the holdfast (*c*_base_). Although this method produced a slightly weaker fit, it provided a more robust and repeatable measure under the prevailing field conditions.

The predictive equation used was:

(6) $$\ln\;\left(B_{>10\;\mathrm{cm}}\right)=1.866\;\ln\left(Lc_{\text{base}}\right)\;–\;5.925\;\left(R^{2}\;=\;0.901,\;p\;<\;0.01,\;n\;=\;217\right),$$

For individuals measuring 2–10 cm in length, we applied a separate linear relationship between fresh weight (FW) and length:

(7) $$\ln\;\left(B_{210–\mathrm{cm}}\right)=1.589\;\ln\left(L\right)\;–\;3.913\;\left(R^{2}\;=\;0.761,\;p\;<\;0.001,\;n\;=\;69\right)$$

For each annual visit to the study site, we measured *L* and *c*_base_ of all marked individuals as well as *L* of the small individuals (2–10 cm) and used Equations 6 and 7 to estimate biomass. The total biomass (kg FW · m^−2^) of each plot was computed as the sum of the estimated biomass of the marked individuals and the unmarked individuals measuring 2–10 cm. Juveniles <2 cm were excluded from biomass estimates, as they were a negligible component compared to the adult population.

The biomass of each marked individual reflects the net outcome of vegetative growth—through apical extension of shoot tips and the formation of new shoots developing from the holdfast or as lateral branches—offset by loss of fragments or entire shoots. Consequently, year‐to‐year changes in standing biomass within plots represent the net balance of these processes. A minimum estimate of annual net biomass production was therefore calculated for each plot as the sum of biomass increments of marked individuals that showed a net increase in size from one year to the next. Individuals that experienced a net loss in size were excluded from this calculation to provide a conservative estimate, focused solely on individuals with net increases in biomass and maintaining methodological consistency across years. This approach did not account for lost production in the form of shoot fragments or entire shoots, which may occur between samplings.

#### Growth

Apical growth of *Ascophyllum nodosum* was quantified by measuring the distance between the base of the youngest and second youngest annual bladder as a proxy for the annual length growth of the tips (Marbà et al., 2017). The biomass of this segment was used as a proxy for the annual biomass growth of the tips. Twenty replicate tips—one from each of 20 randomly selected shoots—were harvested in the mid‐tidal zone outside of the permanent plots during the annual visits. After measuring the length, the tips were dried to constant weight at 60°C and weighed, and the average tip weight was calculated. It should be noted that tip growth estimates are conservative because older parts of the frond may also add to the overall individual biomass growth (Lauzon‐Guay et al., 2022).

#### Carbon and nitrogen contents and stocks

The dried *Ascophyllum nodosum* tips were ground and analyzed for nitrogen (N) and carbon (C) content (*n* = 3) using an elemental analyzer (Flash 1112) coupled to an isotope ratio mass spectrometer (Thermo Fisher Scientific). Analyses were done for the period of 2013–2018.

#### Statistical analysis

Differences in measured parameters among years in the 10 *Ascophyllum nodosum* plots were compared using repeated‐measures analysis of variance (ANOVA), followed by the Tukey honestly significant difference (HSD) post hoc test. Prior to the analyses, the data were tested for normality and homogeneity of variances using Shapiro–Wilk's W and Levene's tests. When assumptions for parametric tests were not met, the nonparametric Friedman test for repeated measures was used. No statistical analysis was conducted on mortality and recruitment rates due to several years with zero values. The effect of year on *A. nodosum* tip growth and nitrogen content was analyzed using one‐way ANOVA. Relationships between *A. nodosum* response variables and environmental factors (temperature, time of ice break, ice scouring) were evaluated using linear regression.

All statistics were done using JMP Pro 16 (SAS Institute Inc.).

## RESULTS

### Physical variables

**FIGURE 2:**
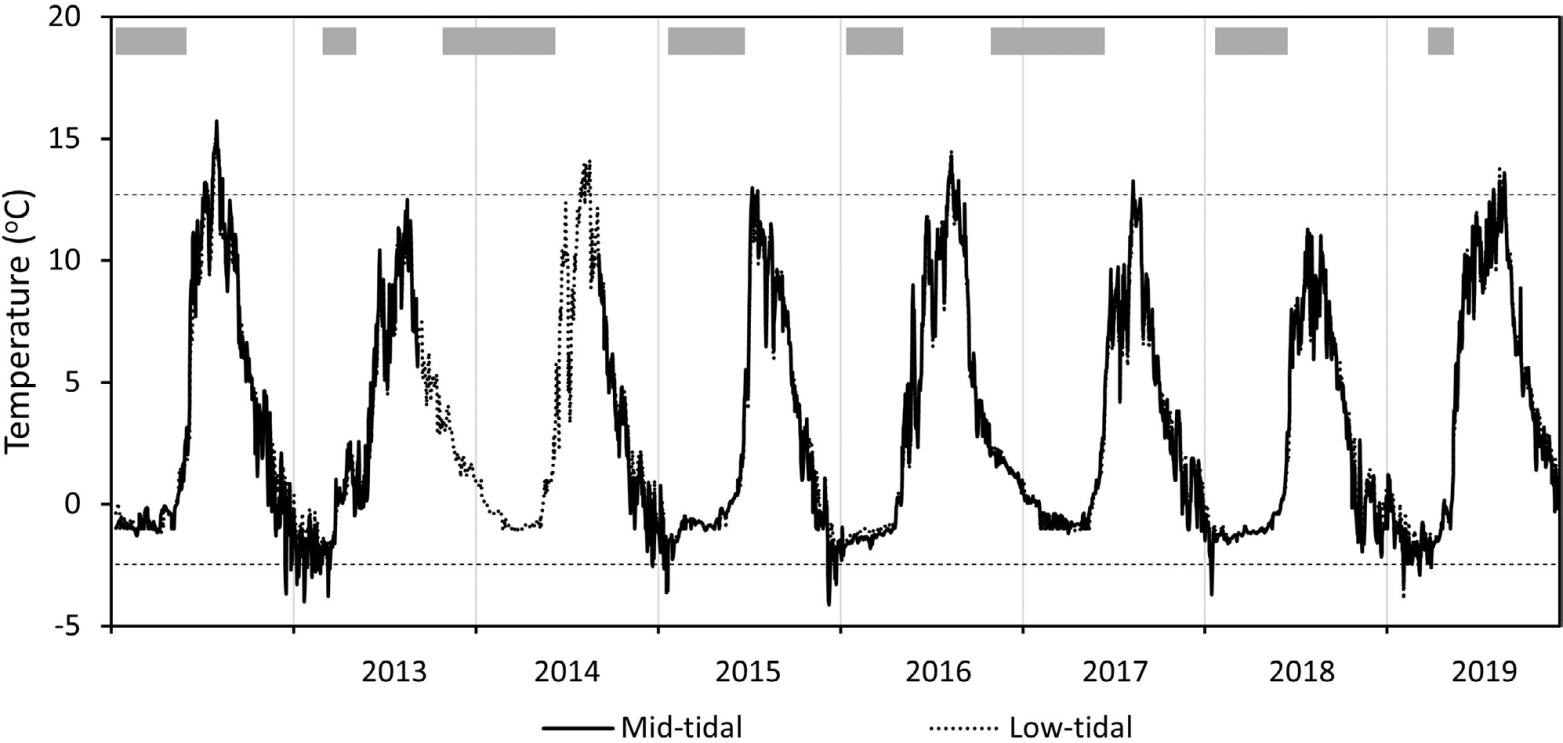
Average daily temperature within the *Ascophyllum nodosum* canopy at mid (MWL) and low (MWL −0.5 m) intertidal level 2012–2019 (data not available for 2013–2014 at MWL). Dotted horizontal lines indicate the 1st and 99th annual temperature percentiles at mid‐intertidal level. Shaded bars show periods of ice cover in the intertidal zone as estimated from daily automatic photography of inner Kobbefjord (see Figure S1).

The seasonal sea ice cover of the intertidal zone ranged from just 2 months during the winters of 2012–2013 and 2018–2019 to 7.5 months during the winters of 2013–2014 and 2016–2017, during which ice on the rocky surfaces developed by mid‐October (Figure 2). Over the 8‐year study period, the ice disappeared between late April and mid‐June. In years of short ice cover (2012–2013, 2015–2016, 2018–2019), ice break‐up occurred more than 1 month earlier than in years with extended periods of ice cover, which given the long spring days, translated to a marked increase in light energy available for *Ascophyllum nodosum* growth.

The ice‐scour index, estimated from the number of bent ice‐screws, varied between 0 in 2019 and 0.5 in 2016 (Table S2). There was no obvious relationship between the period of ice cover and the ice‐scour index. The number of bent screws was unevenly distributed across plots (over the study period, 53% of the bent screws were in two out of 10 plots), suggesting systematic variation in ice scouring intensity within the study area.

The average daily temperature in the *Ascophyllum nodosum* canopy showed marked seasonal variation ranging from −3.5 to 14.7°C (1st to 99th percentiles; Figure 2). Annual median temperature within the *A. nodosum* canopy for 2012–2019 varied between −0.2 and 2.2°C with the highest temperature in years with early break‐up of the ice cover (median temperature versus Julian day of ice‐break: *R* ^2^ = 0.816, *p* = 0.002). Summer temperatures reached a maximum of 11.3–15.7°C in mid‐July–August. The number of days with temperatures above 10°C ranged from 9 days in 2018, the coolest year in the study, to a maximum of 62–63 days in 2012, 2016, and 2019. During winters, the average daily temperature was never lower than −4.1°C. Temperature fluctuations under the ice were small, and the coldest temperatures were measured in years with short periods of ice cover (Figure 2).

For the sake of clarity, the interrelated population responses of *Ascophyllum* are presented variable by variable in the following sections.

### Interannual variation in density of adult and juvenile *Ascophyllum nodosum* individuals

**FIGURE 3:**
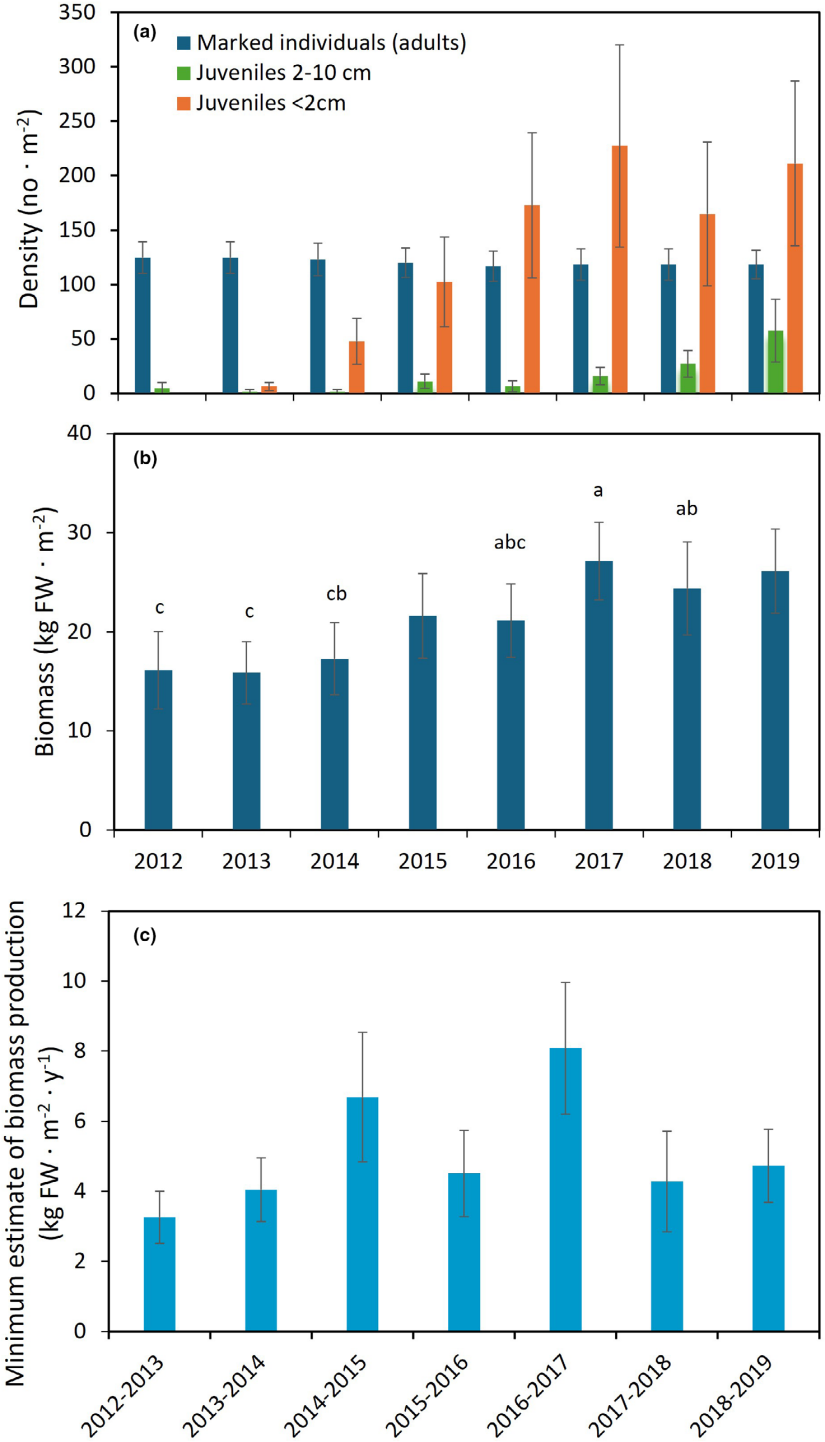
Number of adult (tagged) and juvenile individuals (a), biomass of adult individuals (b) and minimum estimate of annual biomass production (c) in the *Ascophyllum nodosum* population at the mid intertidal zone of Kobbefjord over the period 2012–2019. Different letters indicate significantly different groups according to Tukey's test. Data are means of 10 plots (±*SE*).

The mean density (±*SE*) of adult tagged individuals was nearly constant over time (Friedman test, *p* = 0.707), fluctuating across a very narrow range with a minimum of 116.8 ± 14.0 ⋅ m^−2^ in 2016 and a maximum of 124.8 ± 14.4 ⋅ m^−2^ in 2012 and 2013 (Table 1, Figure 3a). *Fucus vesiculosus* occurred in some of the plots (overall average: 3.2–6.4 individuals · m^−2^) and never exceeded 16 cm in length.

**TABLE 1:** Summary statistics of Friedman Rank test and repeated‐measures ANOVA assessing the effect of year on various parameters of *Ascophyllum nodosum* over an 8‐year period. The parameters analyzed included the density of tagged individuals (adults), juveniles (2–10 cm and <2 cm, respectively), net population growth, total biomass, estimated biomass production, and the median length and age of tagged individuals across 10 permanent plots. Years with zero juveniles (<2 cm) or zero net population growth were excluded from the analyses.

Friedman rank test			
Parameter	*df*	Chi square	*p*
Density (adults ⋅ m^−2^)	7	4.618	0.707
Juveniles 2–10 cm ⋅ m^−2^	7	27.267	<0.001
Juveniles <2 cm ⋅ m^−2^	6	21.0	0.002
Net population growth (ln‐units · year^−1^)	4	1.279	0.845

Repeated‐measures one‐way ANOVA	*df*	*F*‐ratio	*p*
Biomass (g FW · m^−2^)	7	7.052	<0.001
Production (g FW · m^−2^ · year^−1^)	6	2.217	0.055
Median length (cm)	7	1.660	0.1353
Median minimum age (bladders +1)	7	1.790	0.105

The density of new individuals appearing in the plots between censuses varied considerably. No *Ascophyllum nodosum* juveniles <2 cm were observed at the first sampling in 2012, after which they increased in density until reaching a stable level between 164 ± 66.0 and 227.2 ± 93.0 individuals · m^−2^ from 2016 to 2019 (Table 1, Figure 3a). This represented a density that was 65% higher than the adult population density. A similar pattern of increasing density over time was observed for juveniles in size‐class 2–10 cm but with a lag‐phase of 1–2 years. Hence, the number of the larger juveniles varied between 4.8 ± 5.1 and 16.0 ± 8.0 individuals · m^−2^ in the years 2012–2017 and then increased in density to reach a maximum of 57.6 ± 28.8 in 2019 (Figure 3a). There was a highly patchy distribution of juveniles between plots (and within plots) with an overall average density of juveniles <2 cm ranging from 4 to 306 individuals · m^−2^.

### Biomass and minimum estimate of annual biomass production

The total biomass of tagged adults varied significantly during the 8‐year study period (repeated measures ANOVA, *p* < 0.001, Table 1) with a minimum of 16.13 ± 3.92 (*SE*) kg FW · m^−2^ in 2012 and a maximum of 27.13 ± 3.93 (*SE*) kg FW · m^−2^ in 2017. The Tukey's post hoc test indicated a significantly higher biomass during 2017–2019 compared to during 2012–14 (Figure 3b). Although these are coarse biomass estimates, they fall within the range of values for harvested biomass sampled in other years and locations (18.82–34.83 kg FW · m^−2^) within the Nuup Kangerlua fjord system (see Appendix S1 for sampling positions in Table S3 and biomass data, Figure S3).

The minimum estimate of net biomass production, calculated as the sum of biomass gain from all individuals exhibiting an increase in biomass between years, varied 2.5‐fold, ranging from 3.26 ± 0.74 (SE) kg FW · m^−2^ · year^−1^ in 2012–2013 to 8.08 ± 1.88 kg FW · m^−2^ · year^−1^ in 2016–2017 (Figure 3c). This difference across years, however, was not statistically significant (*p* = 0.055, Table 1). The estimated annual production corresponded to a production‐to‐biomass (*P*/*B*) ratio of 0.16–0.38 · year^−1^ and a vegetative biomass turnover time of 2.6–6.3 years.

### Annual recruitment and mortality

**FIGURE 4:**
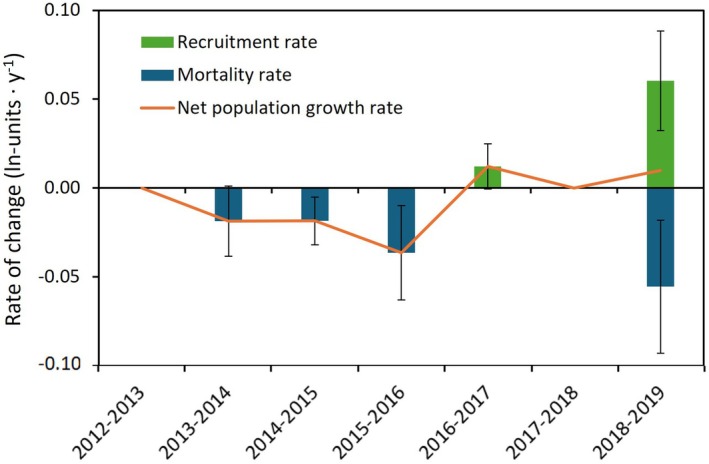
Annual recruitment rates into the adult (tagged) population, mortality rates of adult individuals, and net population growth rate of *Ascophyllum nodosum* at mid‐tidal zone in the inner Kobbefjord between 2012 and 2019. Data are means of 10 plots (±SE).

The loss of adult tagged individuals was low over the 8‐year study period, with an overall average mortality rate (*M*) of 0.019 ± 0.009 (*SE*) · year^−1^ across years (Figure 4), suggesting an individual lifespan (*t*_½_) of 37.5 years. This corresponded to an overall average loss of 1.7% annually. Notably, none of the large, old shoots were lost during the study period. No shoots longer than 35 cm were lost, with lost shoots averaging 26.1 cm in length and having a minimum estimated age of 4 to 8 years.

Recruitment of individuals >10 cm into the adult population occurred only in 2017 and 2019 (Figure 4). The overall average recruitment rate (*R*) of new adults was 0.010 ± 0.009 (SE) · year^−1^, corresponding to an annual recruitment of 1%, which was lower than the mortality rate. Consequently, the population experienced a small decline throughout the study period, with a small negative net population growth rate (*r*) of −0.007 ± 0.007 (SE) · year^−1^ (range: −0.036 ± 0.027 to 0.012 ± 0.013 · year^−1^) across years (Figure 4). Assuming the flux of adult individuals remains constant over time, the halving time of the population would be 93.7 years. However, this estimate includes only the tagged (i.e., adult) individuals and, therefore, underestimates potential population growth, as it does not account for the continuous appearance of small juveniles in the understory.

We were unable to follow the fate of juveniles appearing in the plots between annual counts in late August/early September because they were too small to be marked by our tags. In a previous study of the same *Ascophyllum nodosum* population, juveniles appearing in bare areas 1 year after clearing were less than 2 cm long and exhibited an average growth of 1.7 cm · year^−1^ over following 2 years (Ørberg et al., 2018). Assuming all juveniles <2 cm belonged to the same annual cohort and either died or grew into a larger size class within the following year, the annual appearance of new individuals equaled the number of juveniles <2 cm recorded at each annual census (Figure 3a), resulting in an average input of 116.6 juveniles · m^−2^ · year^−1^ across years. This represented a maximum estimate, because a fraction of juveniles likely remained in the small size‐classes for more than 1 year. A minimum estimate was derived under the assumption that all juveniles <2 cm survived between censuses throughout the 8‐year study period, using the net‐change in density over time. This approach gave an average input of 39.1 new individuals · m^−2^ · year^−1^ across years.

### Length and age class distribution

**FIGURE 5:**
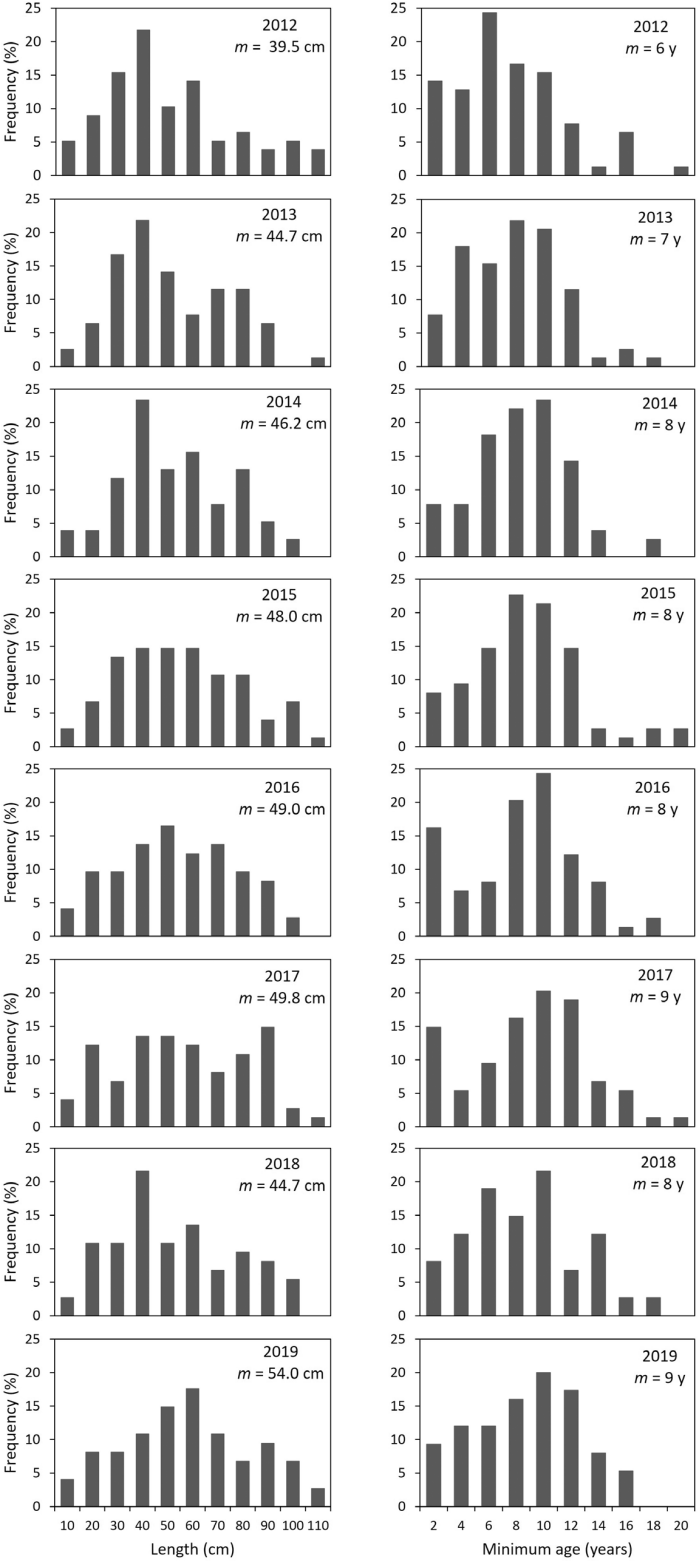
Length (left panel) and minimum‐age (right panel) structure of adult individuals in the *Ascophyllum nodosum* population at the mid‐intertidal zone of Kobbefjord over the period 2012–2019. Minimum age was assessed as the number of bladders +1 on the longest shoot of each individual. Data are pooled from 10 plots (0.25 × 0.25 cm). m: Median length and age; *n* = 74–78 individuals.

The length and age distribution of the adult population was in steady state over years and was dominated by intermediate‐sized individuals. Hence, the median lengths, ranging from 39.5 cm to 54.0 cm, did not vary significantly between years (Table 1, Figure 5). The maximum lengths were between 98.0 and 109.4 cm. The median minimum ages, assessed as the number of bladders along the longest axes of an individual plus 1 year, were 6–9 years. The maximum number of bladders corresponded to ages of 16–19 years (Figure 5). Most marked individuals in the smallest length class (<10 cm) and age class (0–1 bladder) were broken adults. However, considering the increasing pool of juveniles (not included in Figure 5), the overall length and age distributions were skewed toward many small and young individuals (Figure 3a).

**FIGURE 6:**
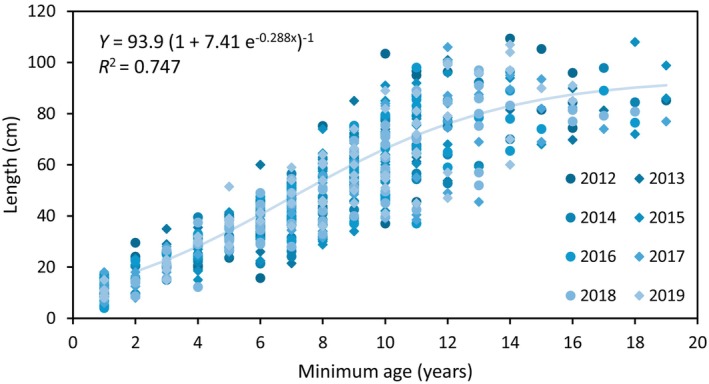
Length in relation to minimum age of adult (tagged) individuals in the *Ascophyllum nodosum* population at the mid‐intertidal zone of Kobbefjord over the period 2012–2019. Minimum age was assessed as the number of bladders +1 on the longest shoot of each individual in 10 plots (0.25 × 0.25 cm). *n* = 74–78 individuals per year. The logistic equation of the fitted line is given. Individuals with no bladder were not included in the regression estimate.

Length was closely related to minimum age (number of bladders) and followed a sigmoid model (*R* ^2^ = 0.747, *p* < 0.001), with an exponential increase in length with increasing number of bladders until approaching a maximum length (asymptote) of 93.9 cm, which was probably set by mechanical abrasion (Figure 6).

### Annual tip growth

**FIGURE 7:**
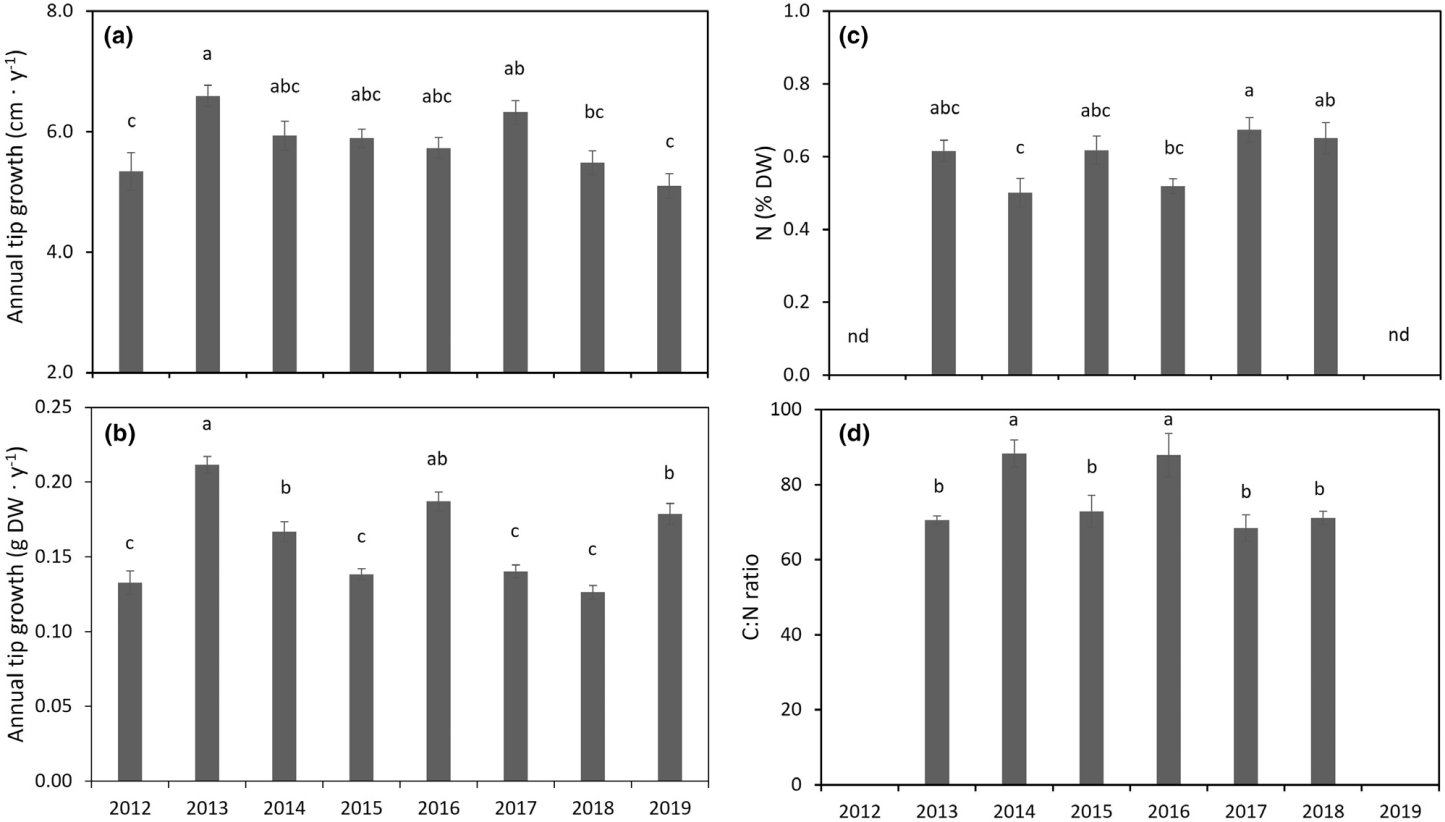
Annual tip growth (average ± *SE*; *n* = 29–39) during the year previous to collection of *Ascophyllum nodosum*, assessed from the length of the youngest complete internode between the first and second bladder (a) and the dry weight of this internode (b). Nitrogen (N) content (average ± *SE*, *n* = 3) in the same internode (c) and the corresponding carbon‐to‐nitrogen ratio (C:N) (d). Different letters indicate statistically significant differences between years.

The annual tip growth of *Ascophyllum nodosum*, assessed from the length of the youngest fully formed internode/segment, was between 5.10 ± 0.20 cm · year^−1^ and 6.60 ± 0.17 cm · year^−1^ with significant differences between years (ANOVA, *F*_7,151_ = 5.827, *p* < 0.001; Figure 7a). Although *A. nodosum* tips increased continuously in length, the stable length distribution of the population indicated that the adult individuals had reached a balance between length gain and loss. The corresponding annual tip biomass growth ranged from 0.126 ± 0.005 g dry weight (DW) · internode^−1^ to 0.211 ± 0.006 g DW · internode^−1^ and also varied significantly between years (ANOVA, *F*_7,151_ = 29.274, *p* < 0.001; Figure 7b).

### Nitrogen content, C/N‐ratio

Tissue N levels of *Ascophyllum nodosum* tips varied significantly between years from 0.50% ± 0.06% to 0.67% ± 0.05% of DW (ANOVA, *F*_5,12_ = 6.150, *p* = 0.005, Figure 7c). This level likely represented an annual minimum, as sampling took place in late summer after the summer growth and reproductive period. Tissue C/N ranged between years from 70.6% ± 0.8% to 88.3% ± 3.0% (ANOVA, *F*_5,12_ = 9.175, *p* = 0.001), showing that the tissues were relatively depleted in nitrogen (Figure 7d).

### Relationships between *Ascophyllum nodosum* variables and environmental variables

The small interannual variations in mortality and recruitment showed no relationship with ice scouring, timing of ice breakup, median temperature, or the number of days with temperatures >10°C (linear regression, *p* > 0.05). Net population growth was also unrelated to those environmental variables.

By contrast, tip biomass growth (but not tip length growth) increased significantly with earlier ice breakup (linear regression, *R* ^2^ = 0.742, *p* = 0.006) and warmer annual median temperatures (linear regression, *R* ^2^ = 0.505, *p* = 0.048) but showed no relationship with the number of days with temperatures >10°C (*p* = 0.633) or with the tissue N content (proxy for nutrient availability; Figure S4). Tip growth was also unrelated to net population growth (linear regression, *p* > 0.05), as the latter reflected the balance between growth and loss.

## DISCUSSION

### Overall stability supported by old productive individuals

This study of *Ascophyllum nodosum* population dynamics in sub‐Arctic settings has documented a stable population with constant density of large, relatively old individuals and a biomass within the upper range recorded for *A. nodosum* populations across its geographical distribution (Åberg, 1992b; Vadas Sr. et al., 2004). Our results suggest that this canopy‐forming species can maintain its ecosystem functions in the sub‐Arctic despite extreme environmental conditions. The biomass turnover, assessed by a *P*/*B* ratio of 0.16–0.38 · year^−1^, was in the lower end of the global range represented by the few other *P*/*B*‐ratios recorded for *Ascophyllum* populations (range 0.2–0.86 · year^−1^; Cousens, 1984; Lamela‐Silvarrey et al., 2012; Vadas Sr. et al., 2004). This aligned with observations of high biomass but low turn‐over in eelgrass (*Zostera marina*) populations at their northern distribution range in Greenland (Olesen et al., 2015) and with the slower turnover of the kelp *Saccharina latissima* in the Arctic compared to further south (Borum et al., 2002).

Our biomass estimate (16–27 kg FW · m^−2^), based on nondestructive sampling, was lower than values reported for nearby areas using destructive harvesting, which amounted to 27–32 kg FW · m^−2^ (Ørberg et al., 2018, Appendix S2: Table S3, Figure S3) and up to 35 kg in another fjord arm of the expansive Nuup Kangerlua. It remains unclear whether this difference reflected methodological uncertainties associated with nondestructive sampling or natural spatial variability, but the comparison suggests that our estimates were conservative. Similarly high, and in some cases even higher, biomasses have been recorded across the species' range, including Maine, United States at 43.5° N (29.9 kg FW · m^−2^, Vadas Sr. et al., 2004), Nova Scotia, Canada at 43.6° N (19.8 kg FW · m^−2^, Sharp, 1987), and Norway at 64–65° N (26 kg FW · m^−2^, Baardseth, 1970). High biomass of *Ascophyllum nodosum* (>24 kg FW · m^−2^) has also been reported from southern Greenland (60° N; Høgslund et al., 2014; Thyrring et al., 2021).

The observed maximum shoot age of at least 16–19 years, assessed from the number of annual bladders along the longest unbroken axis, provided a minimum estimate of longevity. This was because the primary axis of older individuals is often broken, and bladder counts are therefore based on the longest secondary shoot axis formed from the holdfast or as side branches. The modeled average shoot lifespan of 37.5 years, estimated from mortality rates (individual half‐life, Eq2), further underlined that the population was indeed old. The skewed population biomass structure toward large, old individuals aligned with findings from other parts of the species' distribution range (Åberg, 1992a; Araújo et al., 2014). Although shoots of approximately 20 years have been reported in Canada (Lauzon‐Guay et al., 2022), the Greenland population had comparable age, with up to 18 bladders (~19 years) on unbroken shoots, surpassing most previous records (e.g., 10–12 bladders, Baardseth, 1970; 16 bladders, Keser et al., 1981; 17 bladders, Stengel & Dring, 1997). On this basis, the sub‐Arctic *Ascophyllum nodosum* population could be viewed as functionally analogous to “old growth forests” (Olsen et al., 2010, p. 853) or marine trees, concepts used to describe long‐lived, structurally complex macroalgal stands in a seascape context (e.g., Olsen et al., 2010; Thomsen et al., 2024). These populations are relatively large, long‐lived, and stable, although at a very different scale from their terrestrial counterparts. Model projections of *Ascophyllum* populations from the Swedish west coast have suggested a maximum lifespan of up to 50–60 years (based on extrapolated survival data) and that population longevity can be hundreds of years (Åberg, 1992a; Svensson et al., 2009), further supporting this analogy.

The overall stability of the *Ascophyllum nodosum* population and the high shoot ages were supported by the low mortality observed among larger, old shoots. These findings were in accord with previous studies of *A. nodosum* population dynamics elsewhere in its distribution range (e.g., Araújo et al., 2014; Svensson et al., 2009), although Åberg (1992a) reported high mortality during ice winters along the Swedish west coast. Moreover, the relatively substantial productivity that reflected a biomass turnover time of 2.6–6.3 years was essential to compensate for the constant biomass loss. The stable canopy height, despite an annual tip growth of 5–6 cm, underscored a dynamic balance between growth and loss, indicating that the population had reached a steady state. Hence, shoot breakage was being rapidly replaced by regrowth either laterally from side branches or basally from the common holdfast. Apparent biomass stability, or even a slight increase as observed in this study, conceals an underlying flux of organic matter. Although this turnover was modest compared to that of faster‐growing populations further south, it may be crucial for maintaining long‐term canopy persistence in sub‐Arctic environments. In addition to the actively growing, light brown tips at the top of the canopy, the lower layers were dominated by shaded, dark‐green shoots with tips poised to take over if the overlaying branches were lost. These “suppressed” tips have also been highlighted by other studies (Ugarte et al., 2006; Vadas Sr. et al., 2004).

The net biomass production of the Greenland *Ascophyllum nodosum* population was considerable; although our study delivered a minimum estimate (avg. 5.1 kg FW · m^−2^ · year^−1^ or 1.3 kg DW · m^−2^ · year^−1^ and 0.49 kg C · m^−2^ · year^−1^, considering a FW/DW‐ratio of 0.25 and a C‐content of 38% of DW, as measured for the tips). Epidermal shedding was not accounted for in our production estimate, nor in any other estimates we are aware of, but it may constitute as much as 10% of standing biomass every year (Halat et al., 2015); although this value has been contested (Ugarte et al., 2017). Moreover, a complete production estimate should also include the reproductive effort, which we missed, as our annual sampling took place later in summer when most of the receptacles were typically lost. Only the remains of reproductive structures and accumulations of receptacles on the seafloor revealed that extensive reproduction had taken place. The reproductive effort can be considerable, constituting 40%–70% of annual production in Nova Scotia, Canada (Cousens, 1986), 70% at the Swedish west coast (Åberg, 1996), and 29%–71% in Maine, United States (Vadas Sr. et al., 2004), with the effort increasing with the size of individuals (Åberg, 1996).

Despite uncertainties, our productivity estimate for the Greenland population matched the only other known estimate of *Ascophyllum nodosum* productivity from a similar latitude, which was measured from harvested biomass twice per year (1.3 kg DW · m^−2^ · year^−1^ or 0.39 kg C · m^−2^ · year^−1^, (using a C:DW conversion of 0.3), at 65° N in the White Sea, cited in Cousens, 1984). Other productivity estimates, derived using various methods with or without accounting for reproduction, have ranged from 0.013 to 0.93 kg C · m^−2^ · year^−1^ over latitudes from 41 to 53° N, with the highest values from Nova Scotia, Canada, and El Puntal, Spain, both at 43.5° N (compiled by Pessarrodona et al., 2022). The global average net primary productivity (NPP) of intertidal algae (Duarte et al., 2022; Pessarrodona et al., 2022) was only slightly higher than our estimate from Greenland.

A large pool of juveniles supported the recruitment of new adult individuals, compensating for the mortality of the older shoots, thereby contributing to overall population stability. The estimated annual input of juveniles <2 cm was 39–117 individuals · m^−2^, which was comparable to that in the central part of the distribution range (Åberg & Pavia, 1997) and well above the number needed to replace adult losses. The high density of juveniles smaller than 2 cm compared to the density of individuals in the larger size classes suggests substantial early‐stage mortality. Newly settled juveniles of *Ascophyllum nodosum* and other fucoids are susceptible to mechanical disturbance and herbivory (Lubchenco, 1983; Lazo et al., 1994; Dudgeon & Petraitis, 2005; Viana et al., 2014). Once established, however, they may persist for years beneath the adult canopy, growing slowly due to light limitation. This results in an understory of suppressed juveniles that remain in a dormant‐like state until canopy openings allow for faster growth (Ang, 1991; Cousens, 1985). Because our sampling was at the end of the reproductive period (late August/early September), the earliest possible settlement of newly formed zygotes would have taken place in June–July, during the peak of reproduction. Thus, juveniles <2 cm observed in our surveys could have originated from the previous year's reproductive output. However, it remains unclear whether the high density of small juveniles reflected continuous annual recruitment or long‐term persistence. Although our study showed that the population is relatively stable and resilient to typical seasonal fluctuations, due to low mortality and steady vegetative regrowth, recovery following major canopy loss is probably slow. This would be due to the slow growth rate of newly established individuals and thus prolonged period for recruits to reach larger, reproductive size classes (Ørberg et al., 2018).

### Environmental drivers of variability

The overall stability of the population over the 8‐year study period was surprising, given the harsh environmental conditions that included marked seasonal variation in ice cover, ice scouring, and temperature, and the indications of nutrient limitation based on low tissue nitrogen concentrations. The lack of temporal relationships among the population‐level variables (mortality, recruitment, net population growth) and environmental factors demonstrated that the population was resistant and resilient to the levels of environmental forcing during the study. Similarly, at the spatial scale, variation in ice scouring between the plots did not translate to differences in mortality or recruitment. These findings contrast with observations from Sweden, where *Ascophyllum nodosum* populations showed higher mortality in years with ice‐scouring than in years without (Åberg, 1992a). Effects of ice cover and scouring may vary across locations because of differences in exposure. Hence, ice cover in a protected location like our sampling site in inner Kobbefjord may even provide protection for tidal vegetation by forming a stable ice foot that shields the shore from waves and drift ice, while more exposed sites may experience significant damage from scouring (Sejr et al., 2021).

At the level of individual shoots, there was a significant positive relationship between tip growth and both earlier ice break (resulting in more light) and higher average temperature. Since this stimulation of growth did not translate into measurable increases in population biomass, concurrent branch losses must have kept the biomass at steady state. As early ice breakup was correlated with warmer annual median temperatures, it was difficult to distinguish the potential effect of the two. Nonetheless, tip biomass growth was strongly correlated with ice breakup and showed no relationship with the number of days >10°C. This suggests that ice breakup, and the associated increase in light availability, was a stronger driver of growth than temperature. Marbà et al. (2017) also reported that *Ascophyllum nodosum* tip elongation correlated with both temperature and ice cover across latitudes and over time. Latitude effects were also apparent when shoot growth was assessed from age‐length relationships (slope of Figure 6), with our findings from Greenland showing lower growth rates than similar estimates from Galicia, Spain (Viana et al., 2014). The stimulating effect of rising temperature should be seen relative to the optimum temperature for growth for *A. nodosum*, which is about 15°C (Fortes & Lüning, 1980; Kay et al., 2016) and, hence, markedly above the average daily water temperatures in Kobbefjord. Indeed, temperatures exceeding this optimum could hamper *A. nodosum*, as Kay et al. (2016) reported growth reductions at temperatures above 16°C in laboratory experiments.

Tissue nitrogen content, which reflects the balance between nutrient availability and growth, was very low at the time of sampling in late summer. The levels were well below the critical nutrient concentration (1.7% N of DW) and approached minimum values (0.55% N of DW) reported for the fucoid macroalga *Fucus vesiculosus* in Denmark (Pedersen & Borum, 1996). Exceptionally high C/N ratios further underlined the low nutrient status of the tissue. Nevertheless, interannual variation in tissue nutrients at the time of sampling did not correlate with tip growth, suggesting that annual tip growth was not strongly limited by nutrients. The fact that sampling took place each year at the end of the growing season and after sexual reproduction may help explain the low nutrient content, since growth and reproduction may have drained the tissue nutrient pools. Hence, seasonal data from Ireland (51° N) have demonstrated marked seasonal variation in nutrient status, with the lowest levels in autumn (0.89% N of DW; C/N = 46.8), almost three times lower than spring levels (Tabassum et al., 2016). In the Bay of Fundy, Canada, Chopin et al. (1996) also documented about three times lower tissue nitrogen content in August/September (0.74%–0.96% N of DW) than at the beginning of the growth season in March. Similar seasonal patterns were reported in Spain (42.2° N), where July minimum values for *Ascophyllum nodosum* were 0.62% DW and C/N levels reached 80 (Villares et al., 2013). This matches the late‐season levels in this study (%N 0.5%–0.67%; C/N = 70.6–88.3), indicating that the Greenland population was nutrient impoverished by the end of the reproductive season.

In a warmer future with less sea ice, we expect faster growth and turnover rates of the *Ascophyllum nodosum* population, provided nutrient availability is sufficient. Currently, the species grows below its optimum temperature and experiences seasonal shading from sea ice, both of which limit growth. However, standing biomass may not increase proportionally with this enhanced production as it already is at the upper range of reported values. Further biomass accumulation may be constrained by physical factors, since larger fronds are more prone to breakage from waves and ice. Consequently, under future environmental conditions, the population is likely to increase productivity and turnover while maintaining a relatively stable biomass.

### Standing stock and material fluxes to surrounding ecosystems

The high biomass of the dense Greenland *Ascophyllum nodosum* populations represented a standing carbon stock of 1.5–2.6 kg C · m^−2^ and a nitrogen stock of 24–41 g N · m^−2^ (assuming that the carbon and nitrogen content of the tips are representative for the entire biomass). Considering the turnover time of 2.6–6.3 years, the population would support an annual export to the surroundings of about 0.2–1.0 kg C · m^−2^ · year^−1^ and 4–16 g N · m^−2^ · year^−1^. This is a minimum range, since our estimates of the vegetative production were minimum values and the (unquantified) flux of reproductive material was not included. Hence, despite a slower turnover of the population biomass than further south, the sub‐Arctic *A. nodosum* population still supports relatively large fluxes of organic matter to surrounding ecosystems. Part of the exported buoyant biomass will be transported with surface currents, and the fraction that is not mineralized may eventually reach carbon (and nitrogen) sinks in the ocean or on the shore or may support secondary production. A recent study from the Nuup Kangerlua fjord system documented that *A. nodosum*, *Fucus vesiculosus*, and *F. distichus* represented a major part of the exported floating biomass, particularly around ice breaks, and was either retained in the fjord system and surrounding beaches or subjected to long‐term transport (Ager et al., 2023). Atlhough part of the biomass may contribute to carbon sequestration, degradation of accumulated biomass on the shores may also, to some extent, emit greenhouse gases (Björk et al., 2023).

## CONCLUSIONS

In conclusion, our study has highlighted that under sub‐Arctic conditions in Greenland, *Ascophyllum nodosum* populations can maintain relatively stable populations dominated by large, old, and productive individuals and with a dynamic pool of recruits. This, in turn, indicates high resilience and resistance to the level of environmental forces experienced during the past decade. However, major disturbances affecting the survival and vegetative regeneration of larger individuals may have long‐lasting effects on population structure and dynamics and associated habitat functioning. Although the estimated biomass of the population is in range with maximum levels further south, the annual production and turnover are slower. Positive relationships between growth rates and temperature and earlier ice break suggest that turnover of these sub‐Arctic populations will increase in the future provided there is sufficient nutrient supply.

## AUTHOR CONTRIBUTIONS

**Birgit Olesen:** Conceptualization (equal); data curation (equal); formal analysis (equal); funding acquisition (supporting); investigation (equal); writing – original draft (equal). **Núria Marbà:** Conceptualization (equal); data curation (equal); formal analysis (equal); funding acquisition (supporting); investigation (equal); writing – original draft (equal). **Carlos M. Duarte:** Conceptualization (equal); data curation (equal); formal analysis (equal); funding acquisition (supporting); investigation (equal); writing – original draft (equal). **Dorte Krause‐Jensen:** Conceptualization (equal); data curation (equal); formal analysis (equal); funding acquisition (lead); investigation (equal); writing – original draft (equal).

## Supporting information


**Appendix S1.** Regression equations linking *Ascophyllum nodosum* shoot biomass and morphology (Table S1), and the relationships between biomass and morphometric traits used to for non‐destructive estimates of Ascophyllum shoot biomass in permanent plots (Figure S2).
**Table S1.** Regression equations of the form ln(*y*) = a ln(*x*) – b, describing relationships between *Ascophyllum nodosum* biomass (*y*, g FW) and morphometric parameters (x) including 𝐿𝑐𝑚𝑎𝑥 2, *Lc*_base_, 𝐿𝑐_base_ 2, and L. Here L is the shoot length, *c*_base_ is the circumference 2–3 cm above the base, and *c*_max_ is the circumference at the broadest part. Biomass samples from four sites, Inner Kobbefjord at study site and 150 away, Central Kobbefjord and Kapisillit (see Figure 1), were initially analyzed separately. The resulting regression lines using *Lc*_base_ were compared across sites and showed no significant differences between their slopes (ANCOVA, *p* = 0.226–0.811). The data were therefore pooled into a combined dataset for further analysis (*N* = 217).
**Figure S2.** Relationship between biomass (g FW) and morphometric dimensions of *Ascophyllum nodosum* individuals in the mid‐intertidal zone of inner and central Kobbefjord and Kapisillit. (A) Relationship between biomass of individuals longer than 10 cm (*n* = 217) and Lc. (B) Relationship between biomass of individuals 2–10 cm (*n* = 69) and L. L: length, c: circumference of the thallus 2–3 cm above holdfast. Note the logarithmic scale on both.

**Appendix S2.** Additional sampling sites with coordinates and sampling effort (Table S3), and corresponding data on *Ascophyllum nodosum* stand structure and biomass (Figure S3) in Nuup Kangerlua, Greenland.
**Table S3.** *Ascophyllum nodosum* sampling sites in Nuup Kangerlua (2009–2012) including geographic position, quadrat size, and number of quadrats harvested. Samples from Inner Kobbefjord were collected at the long‐term study site in 2012, and 150 m away in 2010 and 2011.
**Figure S3.** Shoot length (A), density of individuals (B) and total biomass (C) of *Ascophyllum nodosum* at six sampling events in Nuup Kangerlua: Inner Kobbefjord (K–I) in 2010 and 2011, Inner Kobbefjord study site (K‐IS) in 2012, Central Kobbefjord (K‐C) in 2009 and 2011 and Kapisillit in 2011. Bars show means ± *SE* (*n* = 4–5). Different letters indicate statistically significant differences among groups (Tukey's HSD test).

**Figure S1.** Example photos of inner Kobbefjord showing different seasonal conditions: full ice cover in winter (01.01.2014), ice break‐up (21.05.2012), and open water in summer (04.07.2017). Photo source: Greenland Ecosystem Monitoring, https://doi.org/10.17897/4F6C‐XJ50.

**Figure S4.** Relationship between the annual tip growth of *Ascophyllum nodosum* at mid‐tidal depth at the study site in Kobbefjord and (A) the Julian day of ice break‐up above the canopy, (B) the annual median temperature below the canopy, and (C) the number of days with temperatures above.

**Table S2.** Ice scour index (0–1) during winter periods of the monitoring years, based on the proportion of bent ice screws recorded across the 10 permanent *Ascophyllum nodosum* plots in Kobbefjord.
