# Supplementary material for: Long lifespan and substantial biomass production support stable high biomass of Ascophyllum nodosum under interannual climate fluctuations in Greenland
Source: J Phycol. 2025 Aug 25;61(5):1288–305. doi: 10.1111/jpy.70071 (PMC12547644; doi:10.1111/jpy.70071)
Supplement: Supplementary file 1 — Appendix S1. Regression equations linking Ascophyllum nodosum shoot biomass and morphology (Table S1), and the relationships between biomass and morphometric traits used to for non‐destructive estimates of Ascophyllum shoot biomass in permanent plots (Figure S2). Table S1. Regression equations of the form ln(y) = a ln(x) – b, describing relationships between Ascophyllum nodosum biomass (y, g FW) and morphometric parameters (x) including 𝐿𝑐𝑚𝑎𝑥 2, Lc base, 𝐿𝑐base 2, and L. Here L is the shoot length, c base is the circumference 2–3 cm above the base, and c max is the circumference at the broadest part. Biomass samples from four sites, Inner Kobbefjord at study site and 150 away, Central Kobbefjord and Kapisillit (see Figure 1), were initially analyzed separately. The resulting regression lines using Lc base were compared across sites and showed no significant differences between their slopes (ANCOVA, p = 0.226–0.811). The data were therefore pooled into a combined dataset for further analysis (N = 217). Figure S2. Relationship between biomass (g FW) and morphometric dimensions of Ascophyllum nodosum individuals in the mid‐intertidal zone of inner and central Kobbefjord and Kapisillit. (A) Relationship between biomass of individuals longer than 10 cm (n = 217) and Lc. (B) Relationship between biomass of individuals 2–10 cm (n = 69) and L. L: length, c: circumference of the thallus 2–3 cm above holdfast. Note the logarithmic scale on both. [file JPY-61-1288-s003.pdf]

APPENDIX S1. Regression equations linking *Ascophyllum* shoot biomass and morphology (Table S1), and the relationships between biomass and morphometric traits used to for non-destructive estimates of Ascophyllum shoot biomass in permanent plots (Figure S2).

Table S1. Regression equations of the form ln (y) = a ln(x) – b, describing relationships between *Ascophyllum* biomass (y, g FW) and morphometric parameters (x) including Lc_{max}^2, Lc_{base}, Lc_{base}^2, and L. Here L is shoot length, c_{base} is the circumference 2-3 cm above the base and c_{max} is the circumference at the broadest part. Biomass samples from four sites, Inner Kobbefjord at study site and 150 away, Central Kobbefjord and Kapisillit (see Figure 1), were initially analyzed separately. The resulting regression lines using Lc_{base} were compared across sites and showed no significant differences between their slopes (ANCOVA, P = 0.226-0.811). The data were therefore pooled into a combined dataset for further analysis (N=217).

Morphometric parameter	R^2	P	b	SE_b	a	SE_a
Lc_{max}^2	0,981	<0.001	-3.128	0.085	0.916	0.011
Lc_{base}	0,901	<0.001	-5.925	0.222	1.866	0.042
Lc_{base}^2	0,871	<0.001	-4.905	0.233	1.281	0.034
L	0,777	<0.001	-6.054	0.364	2.716	0.099

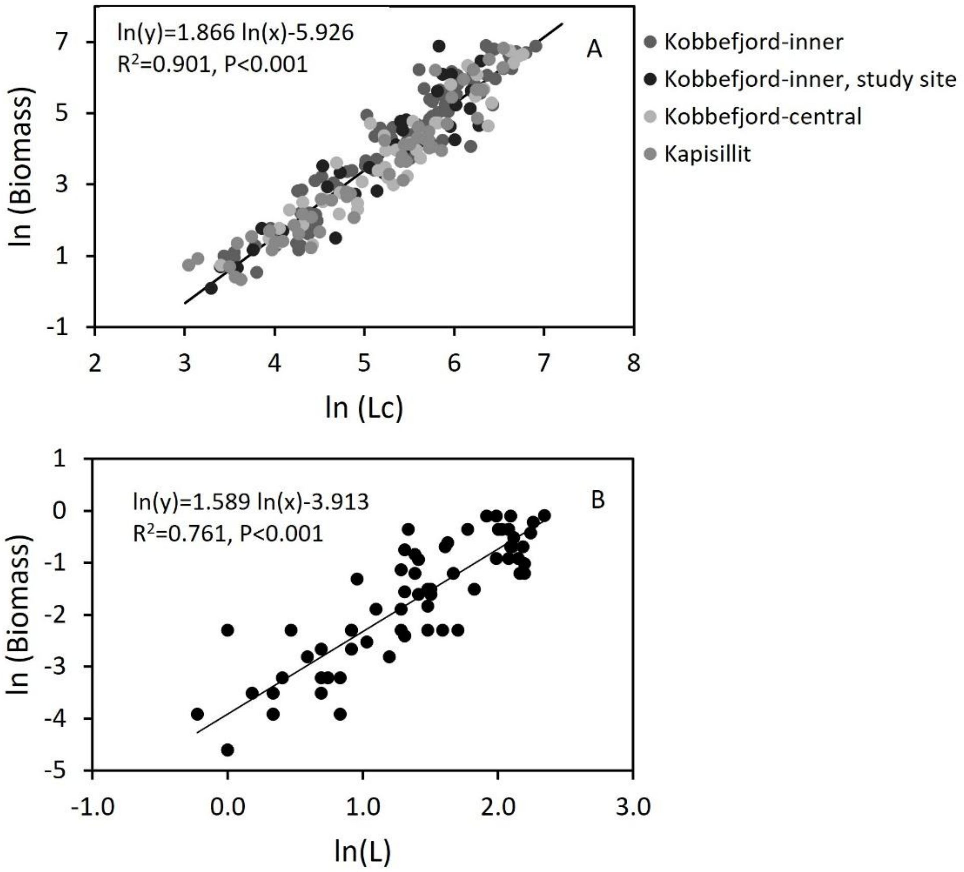

Figure S2. Relationship between biomass (g FW) and morphometric dimensions of *A. nodosum* individuals in the mid-intertidal zone of inner and central Kobbefjord and Kapisillit. A) Relationship between biomass of individuals longer than 10 cm (n = 217) and Lc. B) Relationship between biomass of individuals 2-10 cm (n = 69) and L. L: length, c: circumference of the thallus 2-3 cm above holdfast. Note the logarithmic scale on both axes.
