# Supplementary material for: Long lifespan and substantial biomass production support stable high biomass of Ascophyllum nodosum under interannual climate fluctuations in Greenland
Source: J Phycol. 2025 Aug 25;61(5):1288–305. doi: 10.1111/jpy.70071 (PMC12547644; doi:10.1111/jpy.70071)
Supplement: Supplementary file 2 — Appendix S2. Additional sampling sites with coordinates and sampling effort (Table S3), and corresponding data on Ascophyllum nodosum stand structure and biomass (Figure S3) in Nuup Kangerlua, Greenland. Table S3. Ascophyllum nodosum sampling sites in Nuup Kangerlua (2009–2012) including geographic position, quadrat size, and number of quadrats harvested. Samples from Inner Kobbefjord were collected at the long‐term study site in 2012, and 150 m away in 2010 and 2011. Figure S3. Shoot length (A), density of individuals (B) and total biomass (C) of Ascophyllum nodosum at six sampling events in Nuup Kangerlua: Inner Kobbefjord (K–I) in 2010 and 2011, Inner Kobbefjord study site (K‐IS) in 2012, Central Kobbefjord (K‐C) in 2009 and 2011 and Kapisillit in 2011. Bars show means ± SE (n = 4–5). Different letters indicate statistically significant differences among groups (Tukey's HSD test). [file JPY-61-1288-s005.pdf]

Appendix S2. Additional sampling sites with coordinates and sampling effort (Table S3), and corresponding data on *Ascophyllum nodosa* stand structure and biomass (Figure S3) in Nuup Kangerlua, Greenland

Ascophyllum biomass was harvested from quadrats at multiple sites in Kobbefjord and Kapisillit between 2009 and 2012. All samples were analysed for total biomass (g FW), and in 2011 and 2012, the number and dimensions of individuals longer than 10 cm were also recorded (Table S3).

The average shoot length of *Ascophyllum* individuals larger than 10 cm ranged from 38.5 and 50.9 cm and did not differ significantly between the four sampling sites (ANOVA, $F_{3,234}$=0.632, P=0.595) (Fig. S3). In contrast, the density varied significantly between sample sites (ANOVA, $F_{3,39}$=3.867, P=0.016) with the population from Kapisillit showing nearly twice the density (201.7 ind. m^{-2}) compared to central Kobbefjord (114.0 ind. m^{-2}). *Ascophyllum* biomass was not significantly affected by site or year (ANOVA, $F_{5,46}$=1.293, P=0.283), ranging from 18.8 to 20.7 g FW m^{-2} in central Kobbefjord to 34.8 g FW m^{-2} in Kapisillit (Fig. S3). The overall average biomass across all sites and years was 27.2 ± 2.9 (SE) g FW m^{-2}.

Table S3. *Ascophyllum* sampling sites in Nuup Kangerlua (2009-2012) including geographic position, quadrat size, and number of quadrats harvested. Samples from Inner Kobbefjord were collected at the long-term study site in 2012, and 150 m away in 2010 and 2011.

Site	Year	Position	Quadrat size (m^2)	N
Inner Kobbefjord	2010	64°08N; 51°23W	0.0324	4
Inner Kobbefjord	2011	64°08N; 51°23W	0.0625	5
Inner Kobbefjord, study site	2012	64°08N; 51°23W	0.0225	4
Central Kobbefjord	2009	64°10N; 51°29W	0.0452	5
Central Kobbefjord	2011	64°10N; 51°29W	0.0228	5
Kapisillit	2011	64°28N; 50°13	0.0228	5

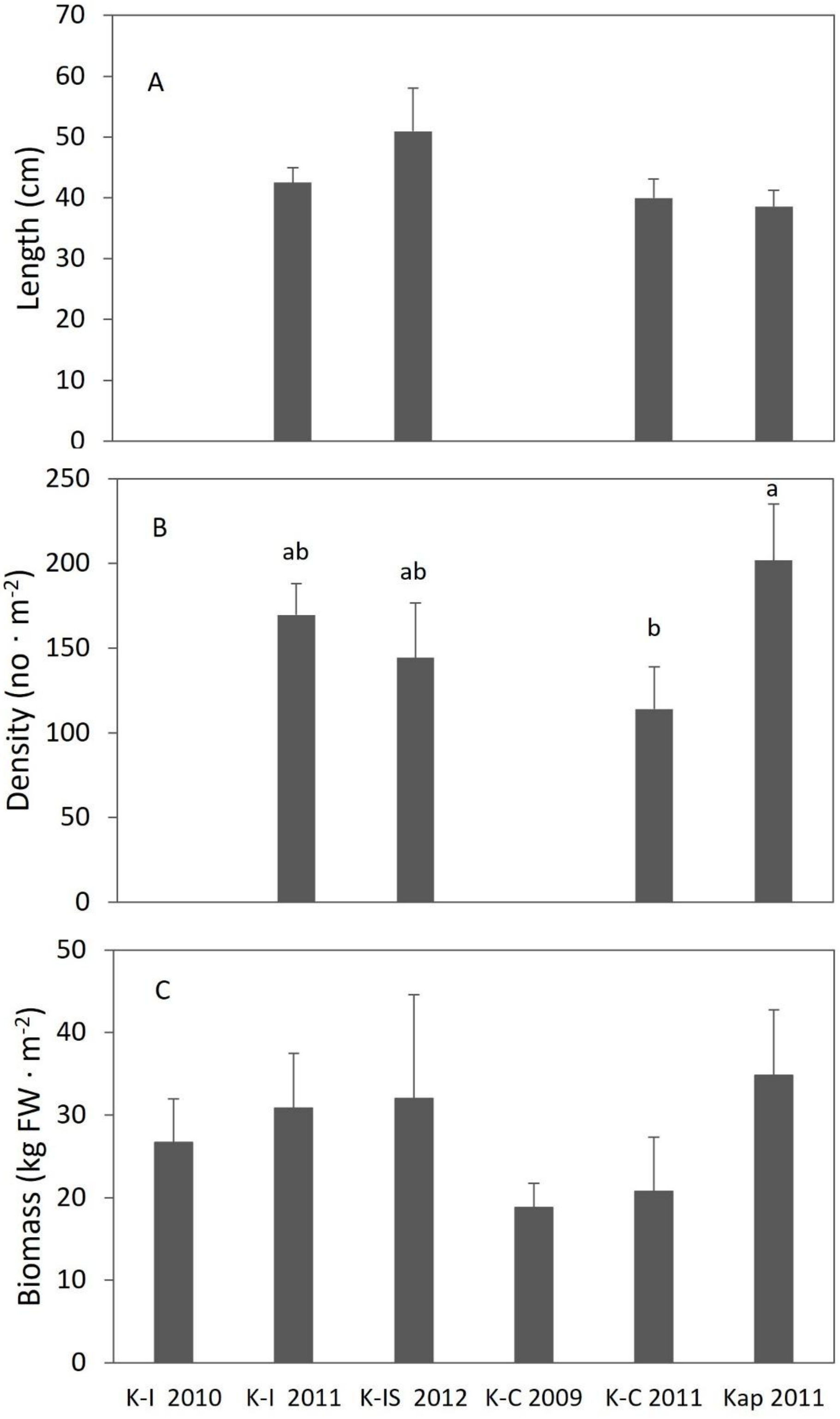

Fig. S3. Shoot length (A), density of individuals (B) and total biomass (C) of *Ascophyllum nodosum* at six sampling events in Nuup Kangerlua: Inner Kobbefjord (K-I) in 2010 and 2011, Inner Kobbefjord study site (K-IS) in 2012, Central Kobbefjord (K-C) in 2009 and 2011 and Kapisillit in 2011. Bars show means ± SE (n = 4-5). Different letters indicate statistically significant differences among groups (Tukey´s HSD test).
