# Supplementary material for: Long lifespan and substantial biomass production support stable high biomass of Ascophyllum nodosum under interannual climate fluctuations in Greenland
Source: J Phycol. 2025 Aug 25;61(5):1288–305. doi: 10.1111/jpy.70071 (PMC12547644; doi:10.1111/jpy.70071)
Supplement: Supplementary file 3 — Figure S1. Example photos of inner Kobbefjord showing different seasonal conditions: full ice cover in winter (01.01.2014), ice break‐up (21.05.2012), and open water in summer (04.07.2017). Photo source: Greenland Ecosystem Monitoring, https://doi.org/10.17897/4F6C‐XJ50. [file JPY-61-1288-s004.pdf]

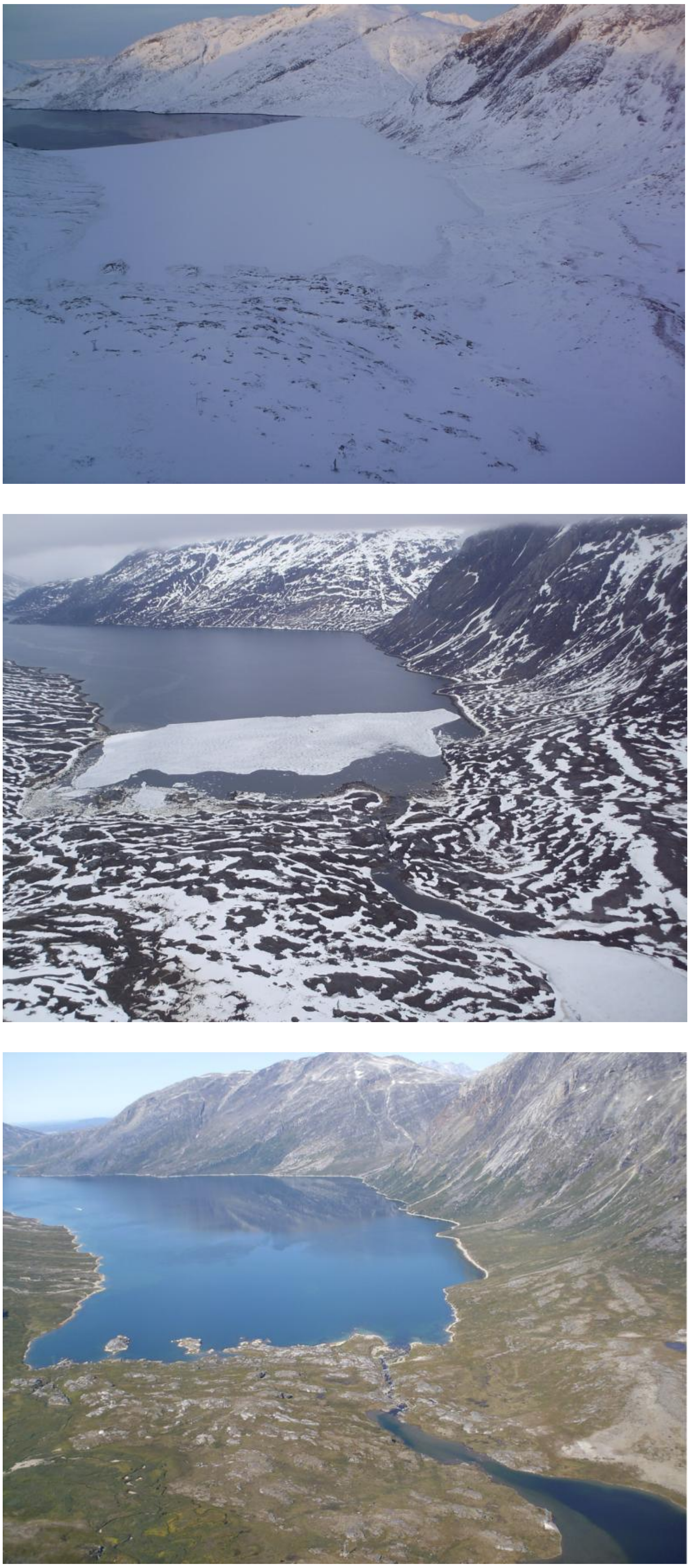

Figure S1. Example photos of inner Kobbefjord showing different seasonal conditions: full ice cover in winter (01.01.2014), ice break-up (21.05.2012), and open water in summer (04.07.2017). Photo source: Greenland Ecosystem Monitoring, https://doi.org/10.17897/4F6C-XJ50.
