# Supplementary material for: Long lifespan and substantial biomass production support stable high biomass of Ascophyllum nodosum under interannual climate fluctuations in Greenland
Source: J Phycol. 2025 Aug 25;61(5):1288–305. doi: 10.1111/jpy.70071 (PMC12547644; doi:10.1111/jpy.70071)
Supplement: Supplementary file 4 — Figure S4. Relationship between the annual tip growth of Ascophyllum nodosum at mid‐tidal depth at the study site in Kobbefjord and (A) the Julian day of ice break‐up above the canopy, (B) the annual median temperature below the canopy, and (C) the number of days with temperatures above. [file JPY-61-1288-s001.pdf]

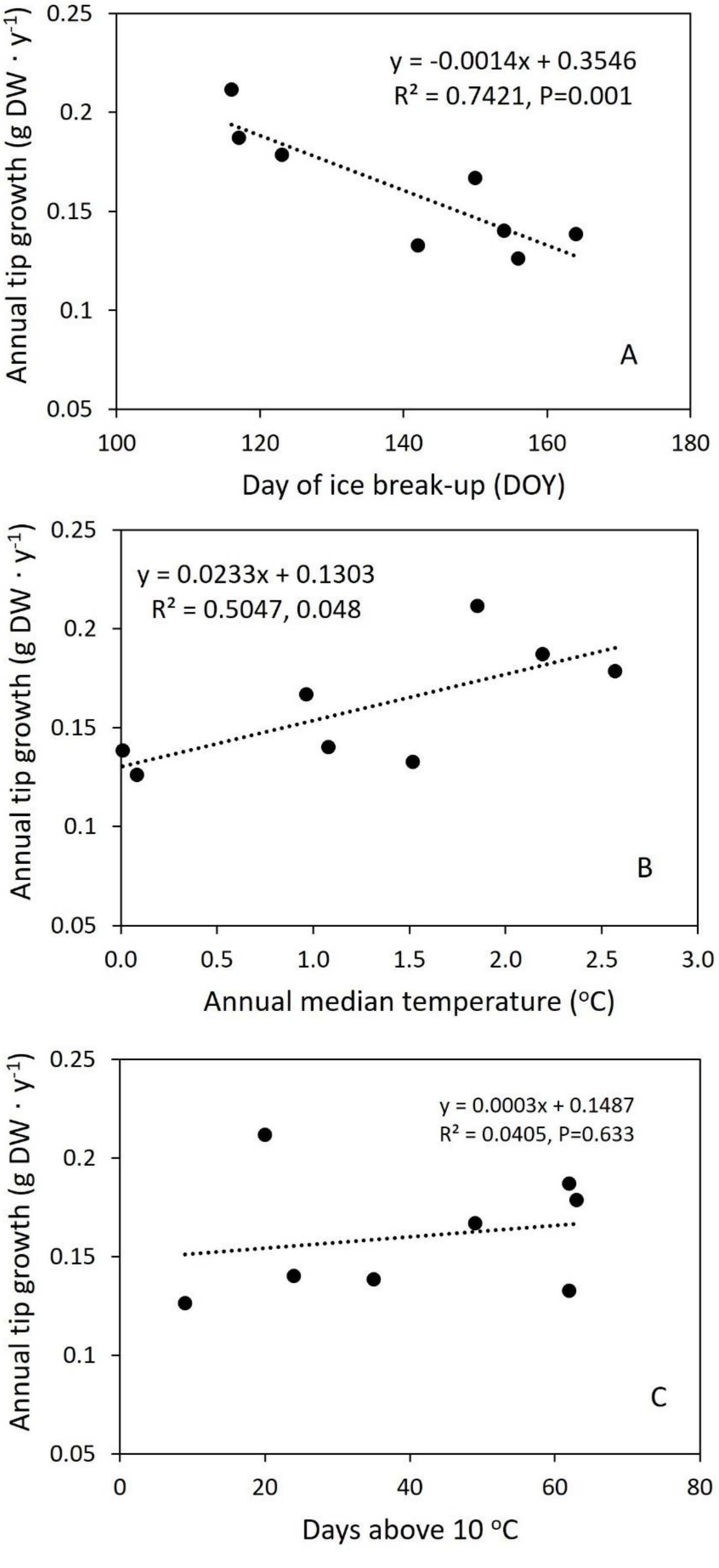

Figure S4. Relationship between the annual tip growth of *A. nodosum* at mid-tidal depth at the study site in Kobbefjord and A) the Julian day of ice break-up above the canopy, B) the annual median temperature below the canopy, and C) the number of days with temperatures above 10°C.
