# Supplementary material for: Long lifespan and substantial biomass production support stable high biomass of Ascophyllum nodosum under interannual climate fluctuations in Greenland
Source: J Phycol. 2025 Aug 25;61(5):1288–305. doi: 10.1111/jpy.70071 (PMC12547644; doi:10.1111/jpy.70071)
Supplement: Supplementary file 5 — Table S2. Ice scour index (0–1) during winter periods of the monitoring years, based on the proportion of bent ice screws recorded across the 10 permanent Ascophyllum nodosum plots in Kobbefjord. [file JPY-61-1288-s002.pdf]

Table S2. Ice scour index (0-1) during winter periods of the monitoring years, based on the proportion of bent ice screws recorded across the 10 permanent *Ascophyllum nodosum* plots in Kobbefjord.

Year	Ice scour index
2012-2013	0.1
2013-2014	0.2
2014-2015	NA
2015-2016	0.5
2016-2017	0.3
2017-2018	0.2
2018-2019	0
